# The Microstructure and Pitting Resistance of 2002 Lean Duplex Stainless Steel after the Simulated Welding Thermal Cycle Process

**DOI:** 10.3390/ma12010070

**Published:** 2018-12-26

**Authors:** Yuanyuan Yang, Yanjun Guo, Yuanyuan Liu, Jin Li, Yiming Jiang

**Affiliations:** Department of Materials Science, Fudan University, Shanghai 200433, China; yangyuanyuan@fudan.edu.cn (Y.Y.); 13110300027@fudan.edu.cn (Y.G.); liuyy@fudan.edu.cn (Y.L.); jinli@fudan.edu.cn (J.L.)

**Keywords:** Lean duplex stainless steel, Gleeble-simulated heat-affected zone, Thermal cycle, Microstructure evolution, Pitting resistance

## Abstract

In this paper, thermal cycles with different heat inputs and cooling rates were investigated for a novel lean duplex stainless steel 2002 using a welding simulation. The microstructure and pitting resistance of the simulated heat-affected zones were studied. With the increasing heat input, the amount and size of the austenite phase both increased, along with a transformation from rods to dendritic structures. The critical pitting temperature (CPT) and the pitting potential (E_pit_) both increased first and then declined as the heat input increased, indicating a strong dependence of pitting resistance on the heat input. For the different cooling rates, the amount of ferrite increased as the cooling rate increased from 0.25 °C/s to 20 °C/s. The CPT and E_pit_ both increased with the increasing cooling rates, indicating an improved pitting resistance. The pits initiated preferentially at the boundaries of ferrite and austenite due to the precipitation of M_23_C_6_ in the specimens with different cooling rates.

## 1. Introduction

Duplex stainless steels (DSSs) have attracted much attention for the satisfactory mechanical properties and favorable corrosion resistance, which are thus widely used in chemical, petrochemical, and nuclear industries [1,2,3,4,5]. The excellent performances of DSSs result from the approximately equal amounts of the two phases: the island-like austenite (γ-phase) and the continuous ferrite matrix (α-phase) [6,7]. The development of lean duplex stainless steel (LDSS), with low contents of nickel and molybdenum and the consequently improved hot workability and weldability becomes a significant focus point for researchers [8,9,10]. In recent years, a novel lean duplex 2002 stainless steel has been developed with a composition of 20.53Cr-3.45Mn-2.08Ni-0.31Mo-0.17N. Previous works from our group have stated several promising properties of LDSS 2002 [7,11,12,13]. For instance, the breaking elongation after annealing at 1050 °C can reach to a maximum of 52.7% owing to the transformation-induced plasticity (TRIP) [11].

Duplex stainless steels inevitably go through welding processes before industrial applications. Welding can severely influence the microstructure and local corrosion resistance of the material [14]. In particular, degradation appears in the heat-affected zone (HAZ) where the intermetallic compounds tend to precipitate, and this precipitation is difficult to prevent [7,15]. The thermal cycle of HAZ can be simulated with different parameters such as the heat input and cooling rate. During the thermal cycle, the alloy is always heated to a peak temperature with a short heating time, then held for a few seconds and ultimately cooled to room temperature. An applied heat source is defined as the heat input, which stands for the amount of energy entering the base metal per unit length of weld from a moving heat source [16]. Heat input is the key factor affecting the thermochemical responses and is related to the cooling process [17,18]. The microstructure, mechanical properties, and corrosion resistance of the welded metals strongly depend on the heat input, which affects the distribution of the main alloying elements of Cr, Mo, and N in the two phases. A higher heat input leads to a lower cooling rate, which is beneficial to phase balance while the grains grow larger [16,19]. On the contrary, a high heat input would cause the precipitation of intermetallic compounds [20,21]. Lundquist et al. [22] found that a lower heat input resulted in the formation of Cr_2_N in a ferrite phase, but a higher input could diminish the amount of nitride precipitates via the reformation of austenite. Deng et al. [23] investigated the isothermal aging of UNS S31803 to simulate a welding thermal cycle and found that the specimens were subjected to the precipitation of secondary phases.

The cooling rate is another important parameter in welding. In the actual welding process, the cooling rate is related to the heat input and the thickness of the plate. HAZ can be divided into the high temperature HAZ (HTHAZ: 1350–800 °C) and the low temperature HAZ (LTHAZ: 1050 °C–room temperature) according to Ramirez, A. J [24]. In the HTHAZ, during the aforesaid process of heating and holding, austenite phases transform into ferrite phases, resulting in the coarsened ferrite phases [14,25]. The precipitation of secondary phases is the main concern for the low temperature HAZ. A suitable cooling rate needs to be adopted to ensure the phase balance and reduce the precipitation of detrimental phases. The HTHAZ cooling rate for LDSS 2002 has been investigated in the previous work of our group [15]. A proper cooling rate of 10 °C/s can provide a balance of austenite and ferrite and avoid the precipitation of Cr_2_N. While for the low temperature HAZ, different cooling rates may cause a myriad of precipitates, such as the carbides (M_23_C_6_), nitrides (Cr_2_N), and intermetallic phases (σ, χ), thus affecting the corrosion properties of steel [26,27,28,29,30]. Lv et al. [31] pointed out that for DSS 2205, the increasing cooling rate in LTHAZ led to a more stable passive film, and the most satisfactory phase balance was obtained with a cooling rate of 20 °C/s.

Therefore, it is essential to investigate the influence of different heat inputs and LTHAZ cooling rates on the microstructure and corrosion resistance of LDSS 2002. Then a full range of welding process parameters can be deduced according to the optimum heat input value and LTHAZ cooling rate given in this work. In the present work, the welding thermal cycles with different heat inputs and LTHAZ cooling rates were performed on LDSS 2002 through a Gleeble thermomechanical simulator. The microstructure evolution of the simulated heat-affected zone (HAZ) was investigated using optical microscopy (OM), scanning electron microscopy (SEM), and transmission electron microscopy (TEM). The pitting resistance was evaluated using potentiodynamic polarization and critical pitting temperature (CPT) tests.

## 2. Materials and Methods

### 2.1. Material

The material used in this study is a newly developed lean duplex stainless steel 2002 provided by Baosteel Research Institute, Shanghai, China, and the chemical composition is shown in Table 1.

### 2.2. Simulated Welding Thermal Cycle Process

**Figure 1:**
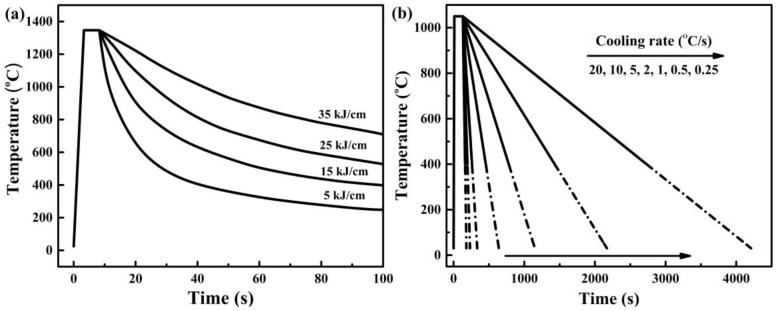
The simulated welding thermal cycles with different (**a**) heat inputs, and (**b**) cooling rates.

The simulation of the welding thermal cycle was carried out using a Gleeble-3800 thermomechanical simulator (Dynamic Systems Inc., Poestenkill, NY, USA). Before the welding simulation, the sample with approximate dimensions of 10.5 mm × 10.5 mm × 55 mm was solution-treated at 1000 °C for 30 min and then quenched in cold water. The thermal cycles with different simulated heat inputs are shown in Figure 1a. The preheated peak temperature in the thermal cycle was 1350 °C and the holding time was 3 s. Then the heat input was set at 5, 15, 25, and 35 kJ/cm, resulting in different cooling processes. To study the effect of cooling rate for the low temperature HAZ on the microstructure and corrosion resistance of DSS 2002, the samples were heated to 1050 °C at a rate of 100 °C/s. After being kept at 1050 °C for 120 s, the specimens were cooled to room temperature at cooling rates of 0.25, 0.5, 1, 2, 5, 10, and 20 °C/s, respectively. The specimens were cut to 10.5 mm × 10.5 mm × 10 mm for microstructure observation and electrochemical tests. For electrochemical tests, the specimens were sealed with epoxy resin. Before all experiments, the specimens were wet ground mechanically with emery papers from 600 to 2000 grit, then polished with a diamond paste of 1.5 μm, rinsed with ethanol and deionized water, and dried in air.

### 2.3. Electrochemical Tests

Electrochemical measurements were conducted using a CHI 660D electrochemical workstation (Shanghai Chenhua Instrument Co., Ltd., Shanghai, China). The tests were carried out with a three-electrode system where a saturated calomel electrode (SCE) acted as the reference electrode, a Pt foil acted as the counter electrode, and the specimens acted as the working electrode. The exposed surface was 1 cm^2^ for the working electrode covered by the 3M tape (3M^TM^ 1600 Vinyl Electrical Tape, 3M Company, Maplewood, MN, USA). All potentials mentioned in the present work were referred to SCE. The test solution was bubbled with pure nitrogen gas (N_2_) to remove the dissolved oxygen (O_2_) before and throughout the test.

The critical pitting temperature (CPT) tests were performed in 1M NaCl solution for its good sensitivity, reproducibility, and efficiency [32,33]. The CPT values were determined through the potentiostatic measurements according to ASTM G 150-99. Prior to the electrochemical tests, the working electrode was cathodically polarized at −900 mV_SCE_ for 120 s. Then, the specimen was allowed to stabilize at an open circuit potential for 10 min. The CPT test was then conducted in 1M NaCl solution at 250 mV_SCE_, and the solution temperature was increased at a rate of 1 °C/min. The current density was continuously recorded with the increasing of temperature. The CPT was defined as the temperature at which the current density reached 100 μA/cm^2^. Potentiodynamic polarization measurements were conducted in the 3.5 wt.% NaCl solution at 30 °C according to ASTM G 59-97. The potential was scanned from −0.8 V_SCE_ in the anodic direction at a scanning rate of 0.1667 mV/s. The potential at which the current reached 100 μA/cm^2^ was identified as the pitting potential (E_pit_). All the experiments were terminated 60 s after the current density increased to 100 μA/cm^2^.

### 2.4. Microstructure Characterization

The microstructure of the specimen was observed by the optical microscopy (OM, Shanghai Microscope Company, Shanghai, China) and scanning electron microscopy (SEM, Philips XL30 FEG, Royal Dutch Philips Electronics Ltd., Amsterdam, Netherlands). Ferrite and austenite phases were distinguished using electrochemical etching in 30 wt.% KOH solution at a potential of 2 V for 10 s before observation. Transmission electron microscopy (TEM, JEOL JEM 2100F, JEOL Ltd., Akishima, Tokyo, Japan) was carried out at 200 kV to examine the secondary phase precipitated in the specimens.

## 3. Results and Discussion

### 3.1. Influence of Heat Input

**Figure 2:**
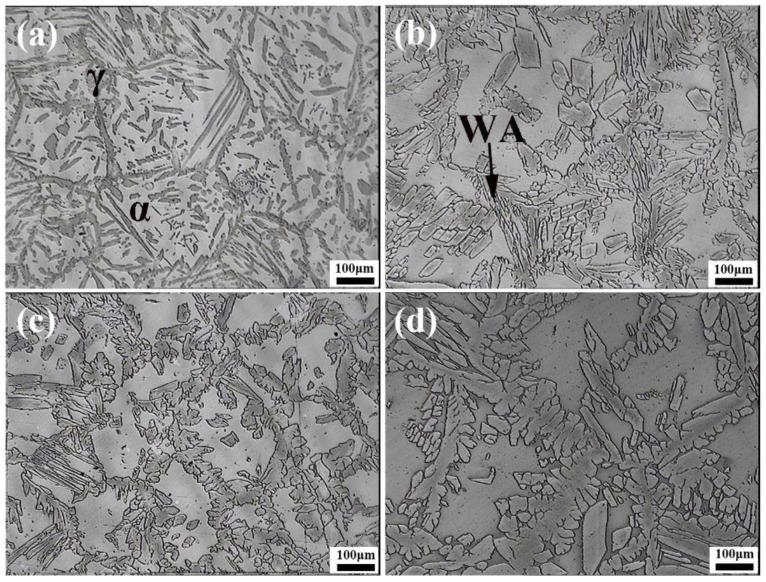
Microstructure of specimens with different heat inputs: (**a**) 5 kJ/cm; (**b**) 15 kJ/cm; (**c**) 25 kJ/cm; (**d**) 35 kJ/cm. α stands for the ferrite phase and γ represents the austenite phase. WA means the Widmanstatten-type austenite.

Figure 2 exhibits the microstructure of LDSS 2002 specimens after the simulated welding with different heat inputs. The dark austenite phase was embedded inside the gray ferrite matrix. As the heat input increased from 5 kJ/cm to 35 kJ/cm, the austenite phase gradually increased and changed from rod-shaped to dendritic. For the heat input of 5 kJ/cm, a small amount of austenite distributed in the ferrite matrix, as shown in Figure 2a. When the heat input increased to 15 kJ/cm as shown in Figure 2b, the Widmanstatten-type austenite (WA) and intragranular austenite (IGA) formed in the ferrite matrix. Meanwhile, both the size and quantity of austenite increased compared with the lower heat input. For the highest heat input of 35 kJ/cm, a large amount of ferrite had transformed into austenite. Figure 2d shows the dendritic austenite phase, which presents a significant increase in the volume content. According to the previous reports, the austenite tends to form at the grain boundary of the ferrite phase [11,32,33]. The phase transformation from ferrite to austenite is controlled by diffusion [25,34,35], which is strongly affected by heat input. The reformation of the austenite phase is determined by a para-equilibrium transformation mechanism where the key process is the diffusion of the elements such as nitrogen. The microstructure of specimens with different heat inputs is similar to the microstructure of specimens with different HTHAZ cooling rates at a range of 1350–800 °C [16]. This was owing to the fact that the heat input was related to the HTHAZ cooling rates and that the higher heat input results in the lower cooling rate. Increasing the heat input will prolong the cooling time, thus promoting the diffusion of austenite-stabilizing elements and ensuring more transformation of ferrite to austenite.

**Figure 3:**
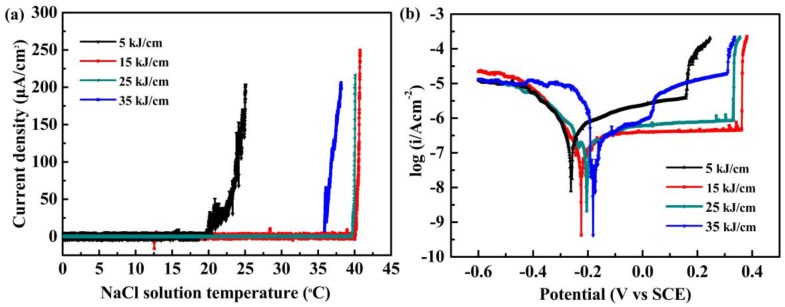
(**a**) The current density plots under 250 mV_SCE_ in 1 M NaCl solution in the critical pitting temperature (CPT) test; (**b**) the potentiodynamic polarization curves in 3.5 wt.% NaCl solution.

Different heat inputs and the induced microstructure evolution may lead to different pitting resistance of the LDSS 2002 heat-affected zone. Figure 3 plots the electrochemical test curves for the LDSS 2002 heat-affected zone with different heat inputs. The CPT curves of current density under 250 mV_SCE_ versus the solution temperature for specimens with different heat inputs are displayed in Figure 3a. The derived CPT value of LDSS 2002 first increased from 24.0 °C to 40.5 °C as the heat input increased from 5 to 15 kJ/cm, and then dropped to 36.8 °C with further increasing of the heat input to 35 kJ/cm. This illustrates that the pitting resistance of LDSS 2002 was improved first and then decreased with the increase of heat input. The highest CPT value of 40.5 °C was obtained for the specimens with a heat input of 15 kJ/cm. There are few current fluctuations in the CPT curve, showing that few metastable pits had nucleated during the tests [36,37].

The pitting potential of LDSS 2002 increased from 0.215 V_SCE_ to 0.370 V_SCE_ as the heat input increased from 5 to 15 kJ/cm, and then decreased to 0.315 V_SCE_ with further increasing of the heat input to 35 kJ/cm (Figure 3b). The specimen with the heat input of 15 kJ/cm presented the largest passivation region and lowest passive current density, indicating a stable passive film formed on the specimen. The results of pitting potential agreed well with the CPT results. The specimens with the heat input of 15 kJ/cm in the welding simulation presented the best pitting resistance.

For the HAZ with a low heat input, it took a short time for the specimens to cool down from the high-temperature region, which could lead to the insufficient diffusion of nitrogen. The saturation solubility of nitrogen in the ferrite phase of lean duplex steel was only 0.05% [9]. The solubility of nitrogen in the ferrite phase also decreased rapidly with a sharp decrease in temperature. The Cr_2_N tended to precipitate in the ferrite phase, resulting in a detrimental effect on the anti-corrosion properties [29,30]. Meanwhile, there is not enough time for nitrogen to spread and generate an austenite phase. When the heat input increased from 5 kJ/cm to 15 kJ/cm, it took more time to cool down so that elements of nitrogen and nickel had sufficient time to diffuse and form the austenite phase, which is beneficial for balancing the ferrite/austenite phases and consequently obtaining the best corrosion resistance of LDSS 2002. When the heat input value was 35 kJ/cm, the proportion of austenite phase became lager. However, the specimens would stay in the intermediate temperature region for an extended period due to a slower cooling rate corresponding to the high heat input. The precipitation of detrimental secondary phases at the boundary of the two phases or inside the ferrite phase during this period could lead to a decreased corrosion resistance of LDSS 2002 [10,38,39,40].

**Figure 4:**
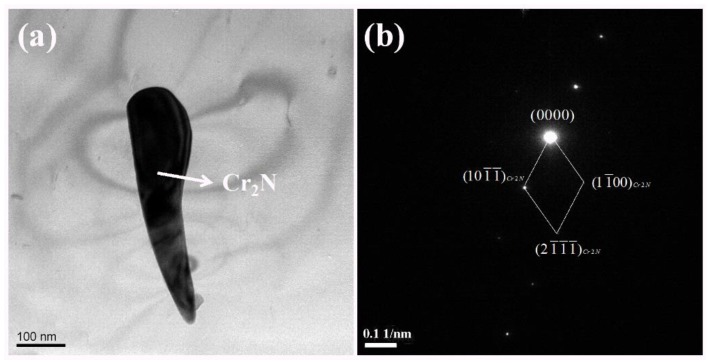
TEM characterization of Cr_2_N in the DSS 2002 specimen with a heat input of 5 kJ/cm: (**a**) a bright-field image; (**b**) diffraction pattern.

Figure 4 presents the TEM characterization of the secondary phase Cr_2_N in the specimen with a heat input of 5 kJ/cm. Figure 4b illustrates a diffraction pattern of nitrides. When the heat input was at a low level, the cooling rate was too fast. The oversaturated nitrogen, which could not diffuse to the two-phase boundary to form an austenite phase, precipitated the slender needle-like Cr_2_N inside the ferrite phase [7,41]. This result reveals that the Cr_2_N has precipitated under the condition of lower heat input, causing the Cr-depleted zone surrounding Cr_2_N. The pits in the electrochemical test may locate at the Cr-depleted zones.

**Figure 5:**
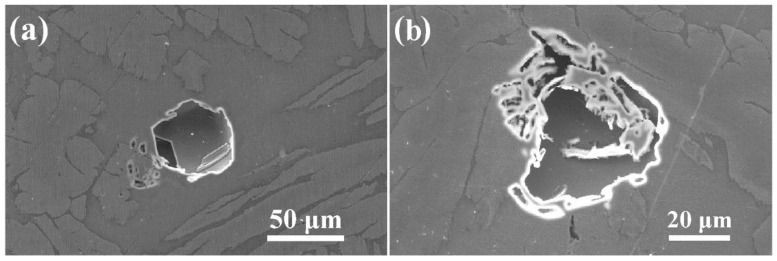
SEM pitting morphologies of specimens with different heat inputs after the CPT tests: (**a**) 5 kJ/cm; (**b**) 15 kJ/cm.

Figure 5 displays the pitting morphologies of the LDSS 2002 specimen with different heat inputs of 5 and 15 kJ/cm after the CPT test. The stable pits mainly distributed inside the ferrite phase when the heat input value was 5 kJ/cm, which may originate from the precipitation of Cr_2_N inside the ferrite (Figure 5a). With respect to the heat input of 15 kJ/cm, the pits stretched across the two phase boundaries and distributed in both the ferrite and austenite phases, indicating the equivalent corrosion resistance of these two phases and consequently leading to the best pitting resistance.

The measured results indicate a clear variation of pitting resistance associated with the increase of heat input. An appropriate heat input value of 15 kJ/cm was significant to prevent the uneven distribution of the ferrite and austenite phases and the precipitation of intermetallic compounds so as to obtain the satisfactory corrosion resistance.

### 3.2. Influence of the LTHAZ Cooling Rate

**Figure 6:**
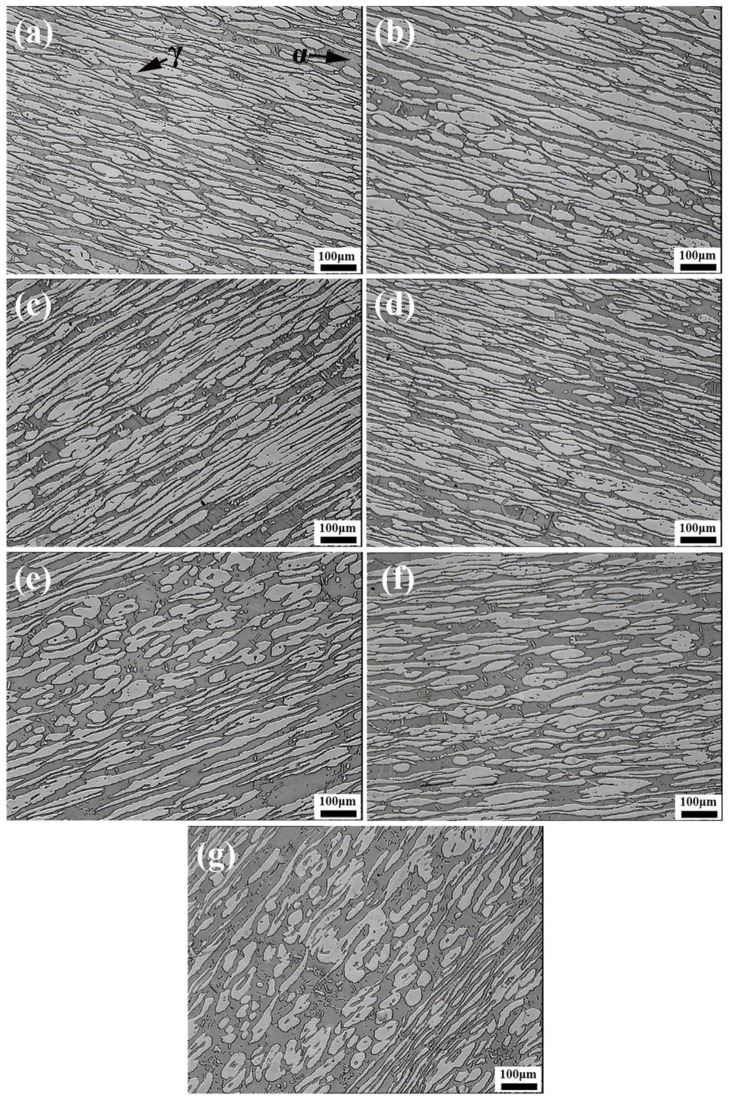
Microstructure of specimens with different cooling rates: (**a**) 0.25 °C/s; (**b**) 0.5 °C/s; (**c**) 1 °C/s; (**d**) 2 °C/s; (**e**) 5 °C/s; (**f**) 10 °C/s; (**g**) 20 °C/s.

For the low temperature heat-affected zone, different cooling rates mainly caused the formation of detrimental secondary phases and influenced the phase balance. Figure 6 shows the microstructure of the specimens with different LTHAZ cooling rates. It can be seen that the ratio of ferrite to austenite in LDSS 2002 sample changed. The amount of ferrite phases increased while the amount of austenite phases decreased, indicating the transformation of austenite to ferrite with the increase of cooling rate. It is obvious that when the cooling rates were 5, 10, and 20 °C/s, the ferrite phase increased and the austenite grains also coarsened compared with the microstructure of specimens with the cooling rates of 0.25, 0.5, 1, and 2 °C/s. This was caused by the longer stay of the specimens in the relatively high-temperature zone due to the slower cooling rates during the LTHAZ cooling process.

**Figure 7:**
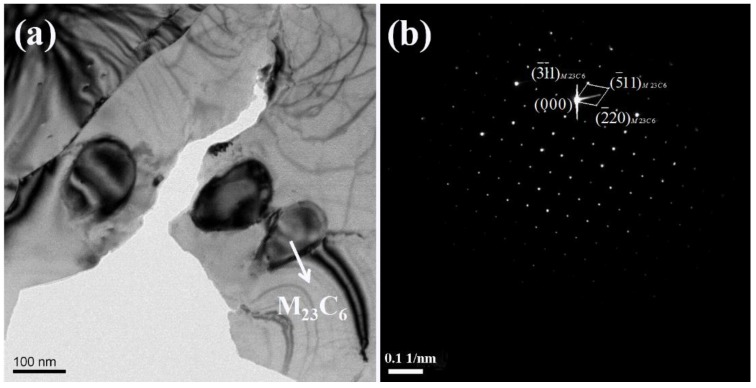
TEM Characterization of M_23_C_6_ phase in the DSS 2002 specimen with a cooling rate of 0.25 °C/s: (**a**) a bright-field image; (**b**) diffraction pattern.

During the cooling process, the LDSS 2002 specimen stayed in the intermediate temperature region for a relatively short time when a fast cooling rate was adopted, and the elements of C, N, and Cr did not have enough time to diffuse to form harmful secondary phases, which was beneficial to the corrosion resistance. Known from the phase fractions in the thermodynamic equilibrium calculated using Thermo-Calc (Thermo-Calc Software, Solna, Sweden) for the LDSS 2002 [11,13], the detrimental secondary phase of M_23_C_6_ would precipitate at the temperature range of 850 to 500 °C. A lower cooling rate means a longer time staying in this temperature range, resulting in the precipitation of the secondary phase. To verify whether the LDSS 2002 specimen had secondary phase precipitations with low cooling rates, the microstructure of the 2002 specimen cooled down from 1050 °C to room temperature with a cooling rate of 0.25 °C/s was characterized using TEM as shown in Figure 7. It confirmed the precipitation of M_23_C_6_ in the sample with a cooling rate of 0.25 °C/s, which has also been reported before [42,43,44]. The specimens were maintained in the intermediate temperature region for a long time because of the much lower cooling rate during the cooling process, which was beneficial to the diffusion of C and N from austenite phase and the diffusion of Cr from ferrite phase to the two-phase boundary, thus forming the harmful secondary phases.

**Figure 8:**
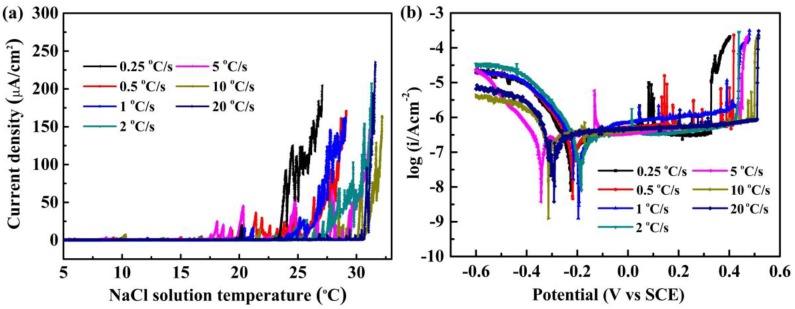
(**a**) The current density curve in the CPT tests under 250 mV_SCE_; (**b**) the potentiodynamic polarization curves in the 3.5 wt.% NaCl solution for the 2002 specimens with different cooling rates.

The cooling rate influences the precipitation of secondary phases in LDSS 2002 sample, which would also affect the pitting resistance of the specimens. Figure 8a reveals the results of critical pitting temperature (CPT) versus the cooling rates. Figure 8b illustrates the potentiodynamic polarization curves of LDSS 2002 specimens with different cooling rates in 3.5 wt.% NaCl solution. The CPT value increased from 23.3 °C to 30.6 °C as the cooling rate increased. The CPT value reached the maximum with a cooling rate of 20 °C/s, indicating that the best corrosion resistance was obtained with the fastest cooling rate. As shown in Figure 8a, there were some current glitches in the CPT curves, which revealed the unstable passive films. However, for the specimens with a cooling rate of 20 °C/s, there were few fluctuations in the curve, suggesting that the passive film on the surface under this condition was stable. The results of the potentiodynamic polarization tests are consistent with the CPT tests. With an increasing cooling rate from 0.25 °C/s to 20 °C/s, the pitting potential increased from 0.376 V_SCE_ to 0.513 V_SCE._ It appears that the corrosion potential of specimens with the cooling rate of 0.25, 0.5, 1, and 2 °C/s was about 0.2 V_SCE_, while the value of the other three samples was near 0.3 V_SCE_. As seen in the potentiodynamic polarization curves, when the cooling rates were 0.25 and 0.5 °C/s, there were distinct metastable current peaks, which may be caused by the precipitation of secondary phases such as M_23_C_6_. Based on the results above, the specimens exhibited the best pitting resistance at a cooling rate of 20 °C/s.

**Figure 9:**
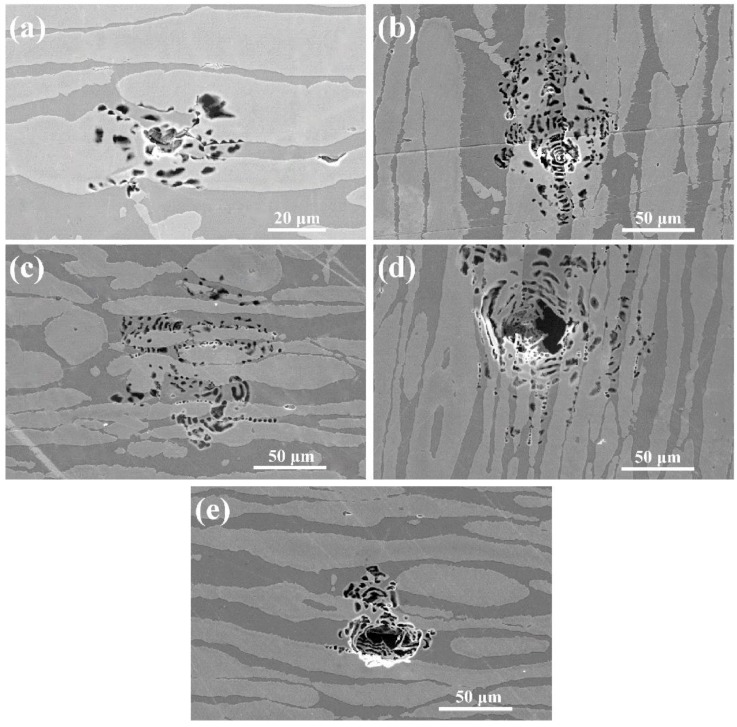
SEM morphologies of specimens with different cooling rates after the CPT tests: (**a**) 0.25 °C/s; (**b**) 1 °C/s; (**c**) 5 °C/s; (**d**) 10 °C/s; (**e**) 20 °C/s.

The SEM morphologies of the LDSS 2002 specimens with different cooling rates after the CPT tests are depicted in Figure 9. All the stable pits present the lace-like topography. According to the literature [45,46], the lace-like morphology illustrates that the hindered diffusion is essential for the formation of stable pits in the early stage of pitting development. As seen in Figure 9, the stable lace-like pits can be found along the ferrite/austenite phase boundaries and preferentially extend into the ferrite phase. If the pits continue to develop, they appear to grow into the austenite phase. This illustrates that the harmful secondary phases of M_23_C_6_ and Cr_2_N may precipitate along the ferrite/austenite boundary during the LTHAZ cooling process, leading to the formation of a Cr-depleted zone. Furthermore, the element Cr in the ferrite phase diffused into the ferrite/austenite boundary, which resulted in the expansion of pits into the interior of the ferrite phase that lacked Cr [47].

## 4. Conclusions

For the purpose of providing scientific guidance and the optimum welding parameters for the manufacturing and welding processes of a novel lean duplex stainless steel 2002, the influence of heat input and cooling rate on the microstructure and pitting resistance in the simulated heat-affected zone of LDSS 2002 were investigated. On the basis of the above experiments, the following conclusions can be drawn:

1. The microstructure of LDSS 2002 changed with different heat inputs. The rod-like austenite phase appeared in the HAZ for a low heat input. With the increase of heat input, the austenite presented as dendritic structures with the increased amount and size.

2. As the heat input rose from 5 kJ/cm to 15 kJ/cm, the CPT increased from 24 °C to 40.5 °C and the E_pit_ increased from 0.215 V_SCE_ to 0.370 V_SCE_. By further increasing the heat input to 35 kJ/cm, the CPT value dropped to 36.8 °C and the E_pit_ decreased to 0.315 V_SCE_, demonstrating that the pitting resistance was enhanced first and later deteriorated. Under the low heat input, the initiation position of pitting altered from the ferrite phase to spanning across both the two phases. For the higher heat input, the pits initiated at the ferrite/austenite interface and subsequently grew into the austenite phase.

3. When the LDSS 2002 specimen was cooled from 1050 °C to room temperature, both the CPT and E_pit_ increased with the increase of cooling rate, indicating that the corrosion resistance had been improved as the cooling rate of LTHAZ increased.

4. During the cooling process, the content of the ferrite phase increased and the austenite decreased with the cooling rate increasing. All the stable pits initiated along the ferrite and austenite boundaries and extended into the ferrite phases. This may be caused by the precipitation of the detrimental secondary phase of M_23_C_6_.

5. For the LDSS 2002 sample in the same condition with that in this research, the optimum heat input and the LTHAZ cooling rate was 15 kJ/cm and 20 °C/s, respectively.

## Figures and Tables

**Table 1:** Chemical composition of DSS 2002.

Element	C	Si	Mn	P	S	Cr	Ni	Mo	Cu	N
wt. %	0.031	0.32	3.45	0.01	0.004	20.53	2.08	0.31	0.34	0.17
